# Cloning and Characterization of the Antiviral Activity of Feline Tetherin/BST-2

**DOI:** 10.1371/journal.pone.0018247

**Published:** 2011-03-29

**Authors:** Aiko Fukuma, Masumi Abe, Yuko Morikawa, Takayuki Miyazawa, Jiro Yasuda

**Affiliations:** 1 Department of Emerging Infectious Diseases, Institute of Tropical Medicine, Nagasaki University, Nagasaki, Japan; 2 Fifth Biology Section for Microbiology, First Department of Forensic Science, National Research Institute of Police Science, Kashiwa, Japan; 3 Kitasato Institute for Life Sciences and Graduate School for Infection Control, Kitasato University, Tokyo, Japan; 4 Laboratory of Signal Transduction, Institute for Virus Research, Kyoto University, Kyoto, Japan; Queensland Institute of Medical Research, Australia

## Abstract

Human Tetherin/BST-2 has recently been identified as a cellular antiviral factor that blocks the release of various enveloped viruses. In this study, we cloned a cDNA fragment encoding a feline homolog of Tetherin/BST-2 and characterized the protein product. The degree of amino acid sequence identity between human Tetherin/BST-2 and the feline homolog was 44.4%. Similar to human Tetherin/BST-2, the expression of feline Tetherin/BST-2 mRNA was inducible by type I interferon (IFN). Exogenous expression of feline Tetherin/BST-2 efficiently inhibited the release of feline endogenous retrovirus RD-114. The extracellular domain of feline Tetherin/BST-2 has two putative *N*-linked glycosylation sites, N79 and N119. Complete loss of *N*-linked glycosylation by introduction of mutations into both sites resulted in almost complete abolition of its antiviral activity. In addition, feline Tetherin/BST-2 was insensitive to antagonism by HIV-1 Vpu, although the antiviral activity of human Tetherin/BST-2 was antagonized by HIV-1 Vpu. Our data suggest that feline Tetherin/BST-2 functions as a part of IFN-induced innate immunity against virus infection and that the induction of feline Tetherin/BST-2 *in vivo* may be effective as a novel antiviral strategy for viral infection.

## Introduction

Human Tetherin/BST-2 (also referred to as CD317 or HM1.24) was first identified as a cellular restriction factor that blocks the release of HIV-1 in the absence of the viral accessory protein, Vpu [1]. Subsequent studies have shown that human Tetherin/BST-2 also inhibits the release of other retroviruses, filoviruses, arenaviruses, and herpesviruses [1]–[7].

Tetherin/BST-2 is a type II integral membrane protein consisting of an N-terminal cytoplasmic tail, a transmembrane domain, followed by an extracellular domain important for dimerization, and a glycophosphatidyl inositol (GPI) lipid anchor at its C-terminus [8]. The extracellular domain of Tetherin/BST-2 has two putative *N*-linked glycosylation sites, which are highly conserved at the same positions among human, rhesus monkey, dog, pig, rat, and mouse, and orthologs have been identified that are actually glycosylated heterogeneously [8], [9]. Previously, we showed that *N*-linked glycosylation is dispensable for the antiviral activity of human Tetherin/BST-2 against Lassa and Marburg viruses [6]. On the other hand, there are conflicting data regarding the role of *N*-linked glycosylation on the antiviral activity of human Tetherin/BST-2 against HIV-1. Andrew *et al*. reported that *N*-linked glycosylation is not important for inhibition of HIV-1 virus release, while Perez-Caballero *et al*. showed that *N*-linked glycosylation, especially at the second site, is important for the antiviral activity of human Tetherin/BST-2 against HIV-1 [10], [11].

Human Tetherin/BST-2 is constitutively expressed in terminally differentiated B cells, bone marrow stromal cells, and plasmacytoid dendritic cells, and is upregulated in various cell types on treatment with type I and type II interferon (IFN) [12], [13]. Therefore, Tetherin/BST-2 is thought to be involved in antiviral host defense as an innate immunity mechanism. It has also been reported that several viruses encode antagonists, such as HIV-1 Vpu, HIV-2 Env, SIVmac/cpz/gor Nef, Ebola virus GP, and Kaposi's sarcoma-associated herpesvirus (KSHV) K5, which antagonize the antiviral activity of Tetherin/BST-2 [1], [5], [7], [14]–[17].

The cat genome contains an infectious endogenous retrovirus (ERV) named RD-114 [18]. Several feline cell lines including Crandell-Rees feline kidney (CRFK) cells constitutively express infectious RD-114 [19], [20]. Therefore, there is concern regarding contamination by RD-114 in vaccines, as these cells have been used to grow several live attenuated vaccines for pets and cattle. In fact, we recently reported the isolation of an infectious RD-114 in a proportion of live attenuated vaccines for pets [21]. RD-114 is considered to be a polytropic virus, since it efficiently infects feline cells as well as human and dog cells [19], [22]. Although the pathogenicity of RD-114 has not been determined, it has potential risks in that interspecies transmission may induce unpredictable diseases. However, it is very difficult to completely exclude the proviral DNA of RD-114 from cells, as ERVs are usually integrated into multiple loci in the host chromosomes [18].

In this study, to investigate the potential of Tetherin/BST-2 to regulate the production of RD-114 from cells, we cloned and characterized the feline homolog of Tetherin/BST-2 and examined its ability to restrict the release of RD-114 from cells.

## Materials and Methods

### Cells

Human embryonic kidney (HEK) 293T cells, Crandell-Rees feline kidney (CRFK) cells, and QN10S cells were maintained at 37°C in a 5% CO_2_ incubator in Dulbecco's modified Eagle's medium (Sigma, St. Louis, MO) supplemented with 10% fetal bovine serum and penicillin/streptomycin. FL74 cells (feline T-lymphoblastoid cell line) were maintained at 37°C in a 5% CO_2_ incubator in RPMI 1640 medium (Sigma) supplemented with 10% fetal bovine serum and penicillin/streptomycin.

### Cloning of the feline Tetherin/BST-2 gene

The primer TethConF (5′-TCACCATCAAGGCCAACAGC-3′), corresponding to a sequence conserved among Tetherin/BST-2 genes from various species reported to date, was designed to clone feline Tetherin/BST-2. RT-PCR was performed using a PrimeSTAR RT-PCR Kit (Takara, Shiga, Japan), with total RNA extracted from IFN-treated CRFK cells as the template, according to the manufacturer's protocols. Partial cDNA fragments of feline Tetherin/BST-2 with high degrees of identity to human Tetherin/BST-2 were amplified. To determine the initiation site of the coding sequence (CDS) of feline Tetherin/BST-2, 5′-RACE was performed with a Takara 5′-Full RACE Core Set according to the manufacturer's protocols (Takara), using total RNA extracted from IFN-treated CRFK, FL74, or QN10S cells as the template. The feline Tetherin/BST-2 gene was identified. The intact CDS of feline Tetherin/BST-2 was amplified again by RT-PCR, and then cloned into pCDNFL, which was constructed from pcDNA3.1 (Invitrogen, Carlsbad, CA) to express a protein containing a FLAG-tag at the N-terminus [6], [23]. The expression plasmid for feline Tetherin/BST-2 was named pfelTeth-FL.

### Plasmids

The plasmids carrying human Tetherin/BST-2 or HIV-1 Vpu, pTeth-FL or pVpu-Myc, respectively, and the plasmid containing an intact infectious clone of RD-114, pTERD-114, were described previously [6], [24]. The glycosylation mutants of feline Tetherin/BST-2 with asparagine to alanine substitution(s) at position 79 and/or 119, N79A, N119A, and N79A/N119A, were generated from pfelTeth-FL using a QuikChange II site-directed mutagenesis kit (Stratagene, La Jolla, CA).

### Quantification of feline Tetherin/BST-2 mRNA by real-time RT-PCR

To examine the induction of feline Tetherin/BST-2 by IFN, CRFK cells were treated for 24 h in the presence of 100 or 1,000 units/ml IFN-αA/D (Sigma) and then total cellular RNA was extracted from pelleted cells using an RNeasy Mini kit (Qiagen, Valencia, CA). Real-time RT-PCR was performed using the feline Tetherin/BST-2 primers, 5′-GGAGTGTCACGGTGTCACCC-3′ (forward) and 5′-CCTCAATCTCTCCCCGAAGCTC-3′ (reverse), and the 18S rRNA primers, 5′-GACGACCCATTCGAACGTCT-3′ (forward) and 5′-TGCTGCCTTCCTTGGATGTG-3′ (reverse). Amplification was performed with a One-Step SYBR RT-PCR Kit (Takara) according to the manufacturer's protocols using a Smart Cycler II System (Cepheid, Sunnyvale, CA).

### Assay for antiviral activity of Tetherin/BST-2 against RD-114

To examine the antiviral activity of Tetherin/BST-2, 293T cells (2×10^5^) were cotransfected with pTERD-114 (100 ng) and either pTeth-FL or pfelTeth-FL (25, 50, 100, or 200 ng) using Trans-IT LT-1 (Mirus Bio Corp., Madison, WI). Forty-eight hours after transfection, virion-containing culture supernatants were clarified by centrifugation (10,000×*g*; 15 min) and virions were pelleted through a 16.5% sucrose cushion by ultracentrifugation (348,000×*g*; 40 min at 4°C). Cells were lysed with lysis buffer A [25]. Virion- and cell-associated proteins were analyzed by Western blotting using anti-RD-114 Gag antibody, anti-FLAG M2 antibody (Sigma), and anti-actin antibody (Sigma) as described previously [6], [24]. The intensities of the bands for virion- and cell-associated Gag were quantified using a Fuji LAS3000 imaging system (Fuji Film, Tokyo, Japan). The control vector (virus p28^CA^/virus p28^CA^ + cellular p28^CA^ + p68^Gag^) was set to 100%. Virus production was also examined by real-time RT-PCR. Viral RNAs were extracted from pelleted virions using a QIAamp Viral RNA Mini Kit (Qiagen). After DNaseI treatment, real-time RT-PCR was performed using the previously described primers targeting the RD-114 *pol* region [26]. Amplification was performed as described above.

## Results

### Cloning and sequence analysis of feline Tetherin/BST-2

**Figure 1:**
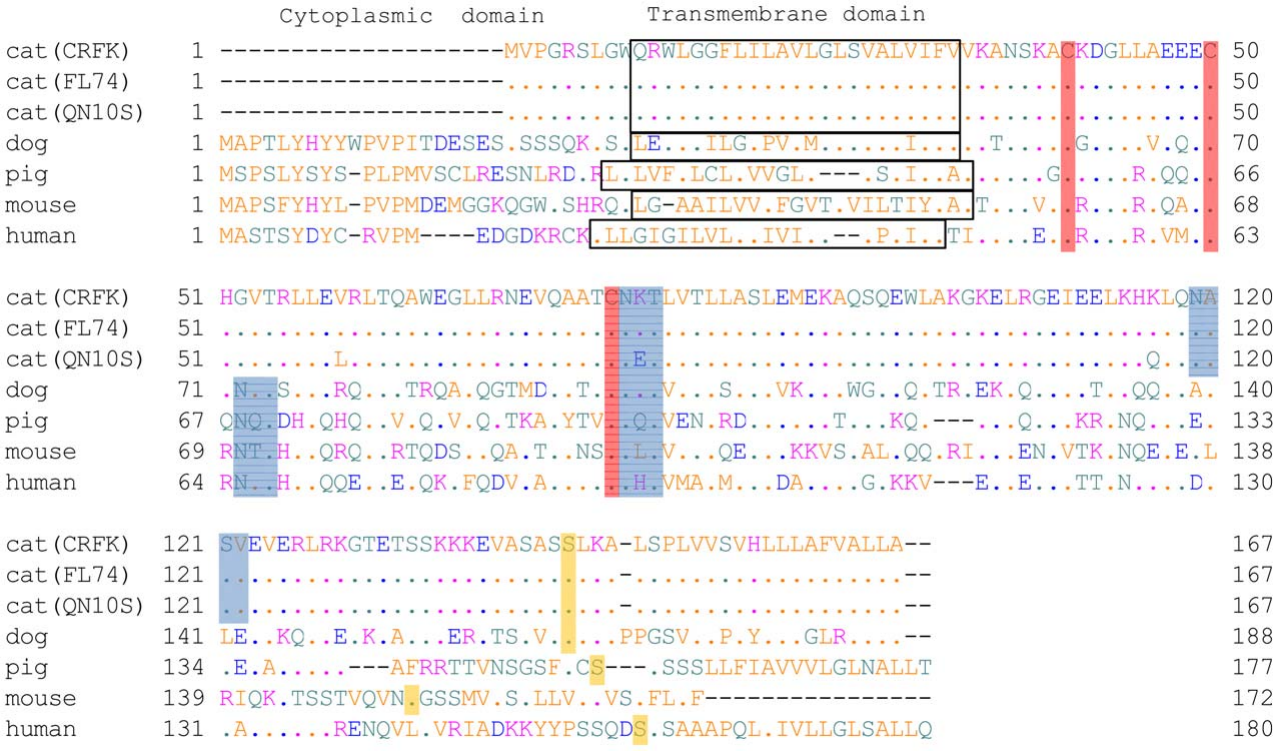
Comparison of the predicted amino acid sequences of cat, dog, pig, mouse, and human Tetherin/BST-2 homologs. Cat (CRFK, FL74, and QN10S cells), dog, pig, mouse, and human Tetherin/BST-2 sequences were analyzed and aligned using GENETYX ver. 8 (GENETYX, Tokyo, Japan). The predicted transmembrane domain is boxed. Three Cys residues in the extracellular domain, which are important for dimerization, are shown with a red background. Two putative glycosylation sites are shown with a blue background. Ser residues of the predicted cleavage site prior to addition of a GPI anchor are shown with a yellow background.

Molecular cloning of complete coding region of feline Tetherin/BST-2 was carried out by RT-PCR and 5′-RACE using RNA extracted from three kinds of feline cell lines, CRFK, FL74, and QN10S cells, treated with IFN. The amino acid sequences of Tetherin/BST-2 from CRFK and FL74 cells were completely identical, while that from QN10S cells was different from those from CRFK and FL74 cells at three positions, 59, 80, and 116 (Figure 1). The nucleotide sequence of the coding region of feline Tetherin/BST-2 and the corresponding protein sequence have been deposited in DDBJ (AB564550). Furthermore, Figure 1 shows the amino acid sequence alignment of Tetherin/BST-2 from cat, dog (GenBank XM_860510), pig (GenBank NM_001161755), mouse (GenBank NM_198095), and human (GenBank NM_004335). The degree of sequence identity between feline Tetherin/BST-2 and those of dog, pig, mouse, and human were 57.7%, 48.7%, 42.5%, and 44.4%, respectively. Three cysteine residues in the extracellular domain, which appear to be important for dimer formation, are conserved among all species. Two putative *N*-linked glycosylation sites are conserved at the same positions among all species other than cat. The glycosylation site in the central region of extracellular domain, N79, is conserved in feline Tetherin/BST-2, while another glycosylation site, N119, is present in the relatively C-terminal region of the extracellular domain of feline Tetherin/BST-2, but not in the region close to the transmembrane domain as in other species. We also found that the N-terminal cytoplasmic tail of feline Tetherin/BST-2 is shorter than those of other species.

### Induction of feline Tetherin/BST-2 in feline cells by IFN

**Figure 2:**
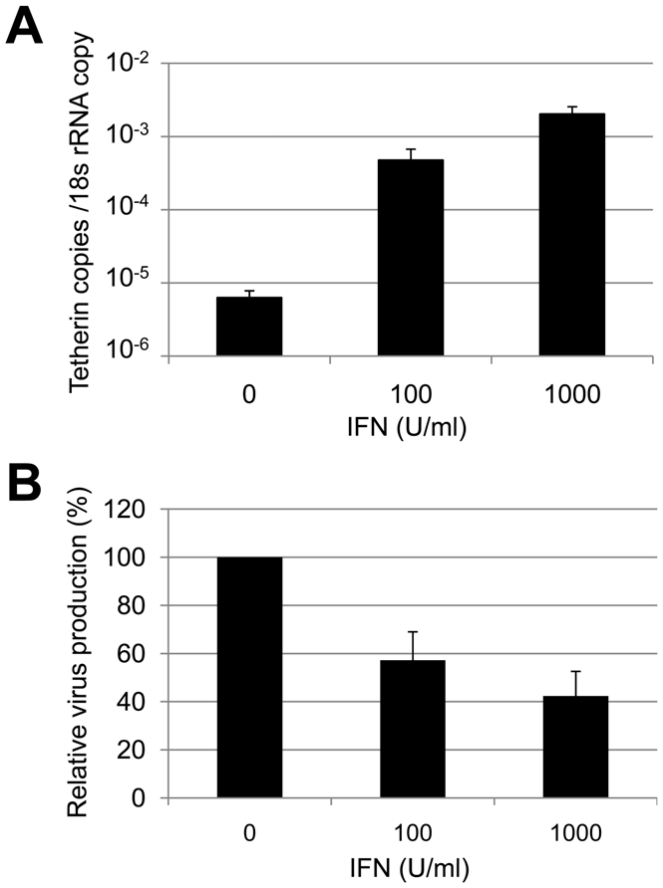
Induction of Tetherin/BST-2 and reduction of RD-114 particle release by IFN treatment of feline cells. CRFK cells were treated with 100 or 1,000 U/ml of IFN-α for 24 h. (A) feline Tetherin/BST-2 mRNA and 18S rRNA were quantified by real-time RT-PCR. The numbers of feline Tetherin/BST-2 mRNA copies were normalized to one copy of 18S rRNA. Histograms represent the averages from three independent experiments (± standard deviation of the mean). (B) RD-114 viral RNA in the supernatant from IFN-treated or untreated CRFK cells were quantified by real-time RT-PCR.

It has been reported that human Tetherin/BST-2 is inducible by type I IFN [1], [12]. To investigate whether the expression of feline Tetherin/BST-2 is induced by type I IFN, we treated CRFK cells with 100 or 1,000 units/ml of IFN-α A/D for 24 h and determined the level of feline Tetherin/BST-2 mRNA by quantitative real-time RT-PCR. Treatment with 100 or 1,000 units/ml of IFN-α induced increases of 75- and 320-fold in feline Tetherin/BST-2 mRNA level, respectively, compared to untreated cells (Figure 2A). CRFK cells constitutively express infectious endogenous retrovirus, RD-114. IFN treatment reduced RD-114 release from CRFK cells with a concomitant increase in feline Tetherin/BST-2 expression (Figure 2B), suggesting that feline Tetherin/BST-2 inhibits release of RD-114 virus particles from cells.

### Antiviral activity of feline Tetherin/BST-2 against RD-114

**Figure 3:**
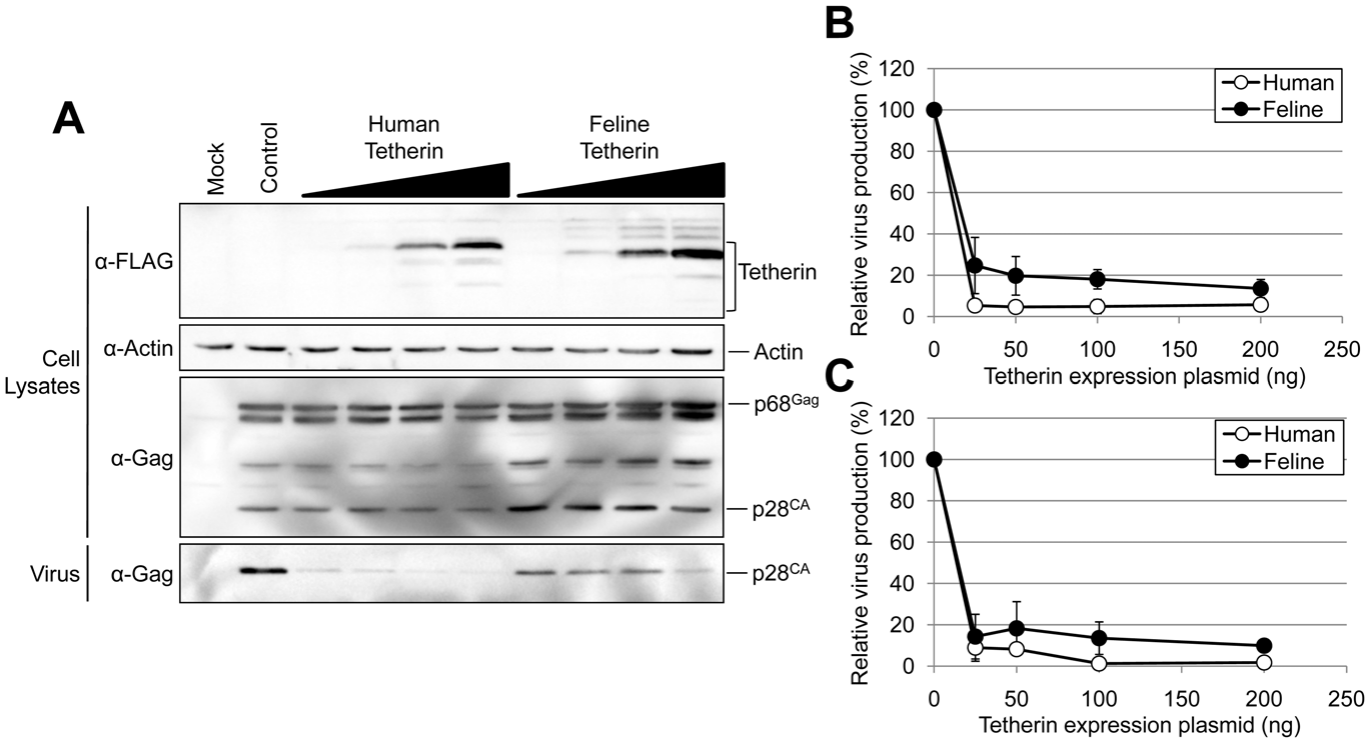
Inhibition of RD-114 particle release by feline Tetherin/BST-2. Both RD-114 vector (100 ng) and the expression vector for human or feline Tetherin/BST-2 containing FLAG-tag (25, 50, 100, or 200 ng) were cotransfected into 293T cells. Cells and viruses were collected at 48 h after transfection, and analyzed by Western blotting (A). The intensities of the bands for virus- and cell-associated Gag were quantified using a Fuji LAS3000 imaging system (Fuji Film) (B). The control vector (virus p28^CA^/virus p28^CA^ + cellular p28^CA^ + p68^Gag^) was set to 100%. Histograms represent the averages from three independent experiments (± standard deviation of the mean). (C) RD-114 viral RNA in the supernatant from cells was quantified by real-time RT-PCR.

To directly examine whether feline Tetherin/BST-2 has inhibitory activity against RD-114 virus release, the expression plasmid for feline Tetherin/BST-2 originated from CRFK cells or human Tetherin/BST-2 was cotransfected with the RD-114 infectious clone into 293T cells and RD-114 production was analyzed by Western blotting and real-time RT-PCR assay. As shown in Figure 3A, dose-dependent reductions in RD-114 release were observed with increasing expression levels of human and feline Tetherin/BST-2. Quantitative analyses of the amounts of RD-114 virions released from cells were also carried out by Western blotting and real-time RT-PCR assay (Figure 3B and C). The inhibition of RD-114 virus release by feline Tetherin/BST-2 was demonstrated by both Western blotting and real-time RT-PCR assay. Antiviral activity of feline Tetherin/BST-2 against RD-114 release was slightly weaker than that of human Tetherin/BST-2, although feline and human Tetherin/BST-2 were expressed at similar levels in cells transfected with the same amounts of each expression plasmid. We also confirmed that feline Tetherin/BST-2 originated from QN10S cells inhibits RD-114 production at the similar level to feline Tetherin/BST-2 originated from CRFK cells (data not shown).

### Importance of *N*-linked glycosylation for antiviral activity

**Figure 4:**
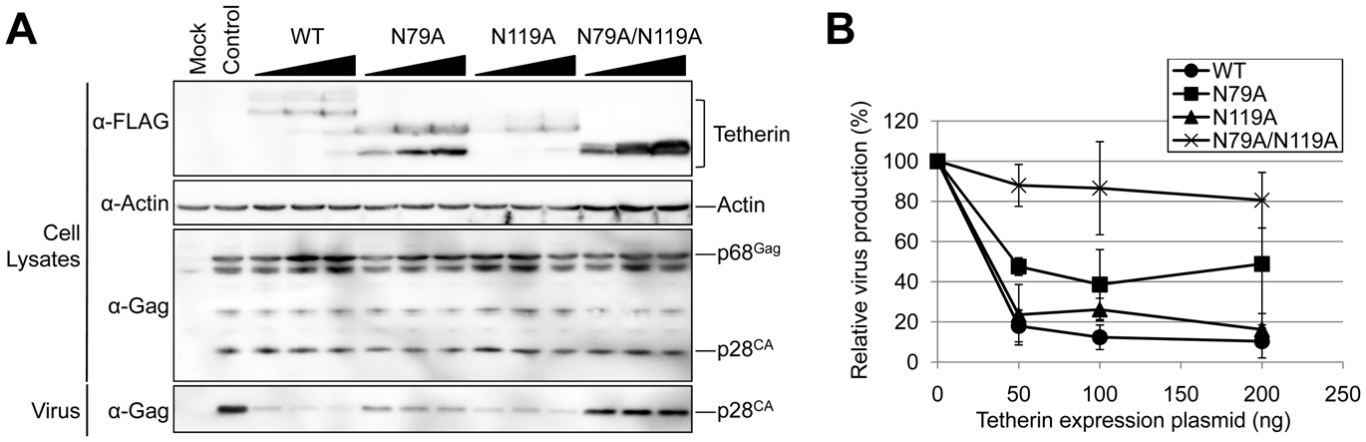
Importance of *N*-linked glycosylation for the antiviral activity. Both RD-114 vector (100 ng) and the expression vector for wild-type or mutant feline Tetherin/BST-2 (50, 100, or 200 ng) were cotransfected into 293T cells. Cells and viruses were collected at 48 h after transfection, and analyzed by Western blotting (A). The intensities of the bands for virus and cell-associated Gag were quantified using a Fuji LAS3000 imaging system (B). The control vector (virus p28^CA^/virus p28^CA^ + cellular p28^CA^ + p68^Gag^) was set to 100%. Histograms represent the averages from three independent experiments (± standard deviation of the mean).

To examine the role of *N*-linked glycosylation of feline Tetherin/BST-2 in its antiviral function, we analyzed the effects of exogenous expression of mutants with a single or multiple mutations in the *N*-linked glycosylation sites (N79A, N119A, and N79A/N119A) on RD-114 production. 293T cells were cotransfected with the RD-114 infectious clone and increasing amounts of the expression plasmid for wild-type or mutant feline Tetherin/BST-2. Wild-type feline Tetherin/BST-2 was detected as triplet bands, while N79A and N119A mutants and N79A/N119A mutant showed double and single band(s), respectively (Figure 4), indicating that the upper, middle, and lower bands of triplet forms corresponded to multiple-, single-, and non-glycosylated forms, respectively. Exogenous expression of the N119A mutant significantly reduced RD-114 virus release as well as wild-type, while the inhibitory activity of the N79A mutant on the RD-114 virus release was lower than those of wild-type and N119A mutant despite the much higher expression level (Figure 4). Furthermore, the N79A/N119A mutant without glycosylation almost completely lost its antiviral activity. In addition, the antiviral activity of the N79A/N119A mutant could not be overcome by increased expression. These results indicated that glycosylation at N119 is not essential for the antiviral activity of feline Tetherin/BST-2, while the loss of glycosylation at N79 or at both N79 and N119 markedly affected its antiviral activity.

### Feline Tetherin/BST-2 is insensitive to antagonism by HIV-1 Vpu

**Figure 5:**
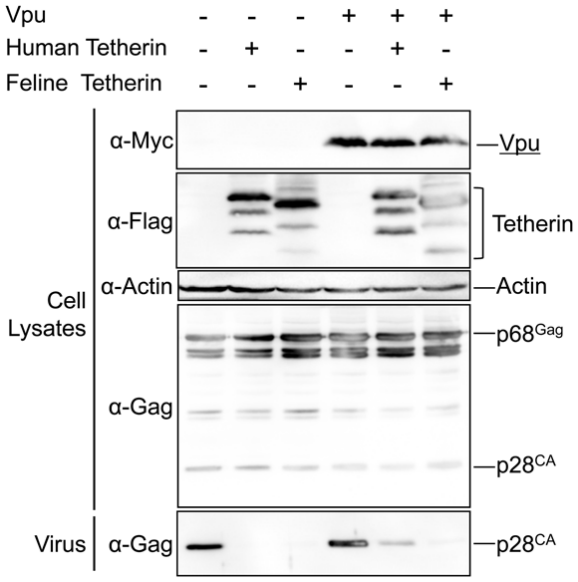
Feline Tetherin/BST-2 is insensitive to antagonism by HIV-1 Vpu. Both RD-114 vector (100 ng) and the expression vector for human or feline Tetherin/BST-2 containing FLAG-tag (200 ng), with or without the expression vector for Vpu containing Myc-tag (1 µg), were cotransfected into 293T cells. Cells and viruses were collected at 48 h after transfection, and analyzed by Western blotting.

HIV-1 Vpu has been shown to antagonize the antiviral activity of human Tetherin/BST-2, but not monkey Tetherin/BST-2. To examine the sensitivity of feline Tetherin/BST-2 to Vpu, pVpu-Myc, which expresses Vpu containing a Myc-tag at the N-terminus, was cotransfected into 293T cells along with the RD-114 infectious clone and the expression plasmid for human or feline Tetherin/BST-2. RD-114 production was analyzed by Western blotting. As expected, Vpu expression partially rescued the RD-114 release reduction by human Tetherin/BST-2, but not that by feline Tetherin/BST-2 (Figure 5), indicating that Vpu has no effect on the antiviral activity of feline Tetherin/BST-2.

## Discussion

In this study, we identified feline Tetherin/BST-2 and demonstrated the antiviral activity of feline Tetherin/BST-2.

The degree of sequence identity between feline Tetherin/BST-2 and those of dog, pig, mouse, and human were 57.7%, 48.7%, 42.5%, and 44.4%, respectively. As compared to the other cellular antiviral factors including APOBEC3 and TRIM5 proteins, the sequence homologies of Tetherin/BST-2 among mammalian species are relatively low. It has been reported that the antiviral activity of Tetherin/BST-2 require the structural features such as an N-terminal transmembrane region, a C-terminal GPI anchor, and a proper coiled-coil formation of extracellular region, but not exact amino acid sequences [11]. It would be the reason why the sequence homologies of Tetherin/BST-2 among mammalian species are relatively low.

As expected, we found that feline Tetherin/BST-2 efficiently inhibited the release of feline endogenous retrovirus RD-114 from cells, although the inhibitory effect of feline Tetherin/BST-2 against RD-114 release was slightly weaker than that of human Tetherin/BST-2 (Figure 3). RD-114 has been reported to be produced as infectious viruses in some feline cell lines and to be present as a contaminant in a proportion of live attenuated vaccines for pets [21]. It is very difficult to completely exclude the proviral DNA of RD-114 from cells, as endogenous retroviruses are usually integrated into multiple loci of the host chromosomes. The induction or exogenous expression of feline Tetherin/BST-2 in these feline cells may be useful as a novel strategy to reduce the risk of the RD-114 contamination into vaccines or biological products.

The sequence analysis of feline Tetherin/BST-2 showed that the N-terminus of feline Tetherin/BST-2 was shorter than those of other species (Figure 1). It has been reported that a dual-tyrosine motif (Y-Y_6–8_) in the cytoplasmic domain of human Tetherin/BST-2 is crucial for clathrin-mediated endocytosis through recruiting AP-1 and AP-2 adaptor proteins [27], [28]. Feline Tetherin/BST-2 may be internalized *via* a different pathway from the others, since feline Tetherin/BST-2 does not have this dual-tyrosine motif in its cytoplasmic domain. These features may have an effect on the weaker antiviral activity of feline Tetherin/BST-2 compared to human Tetherin/BST-2. However, at present, it is not clear whether the short cytoplasmic domain and deficiency of the dual-tyrosine motif are involved in any function of feline Tetherin/BST-2.

Although the expression levels of N79A and N79A/N119A mutants in cells were much higher than those of wild-type and N119A, the loss of glycosylation at N79, but not N119, reduced the antiviral activity of feline Tetherin/BST-2 (Figure 4). Moreover, the loss of glycosylation at both N79 and N119 almost completely inactivated the antiviral activity against RD-114. Glycosylation at N79 is conserved among Tetherin/BST-2 homologs from many species including cat (Figure 1), suggesting that this glycosylation plays an important role in the structure and function of this molecule. In addition, it has been reported previously that *N*-linked glycosylation at the corresponding site of human Tetherin/BST-2 is important for antiviral activity against HIV-1 [11]. On the other hand, glycosylation at N119 is unique in feline Tetherin/BST-2 and appears to be dispensable for the antiviral activity. Although it is not clear how glycosylation of Tetherin/BST-2 affect its antiviral activity, it has been reported that the cell surface expression levels of the glycosylation mutants of human Tetherin/BST-2 are less than that of WT [11]. The lacking of glycosylation signal may affect the intracellular transport of Tetherin/BST-2 and result in loss of the antiviral activity of Tetherin/BST-2.

HIV-1 Vpu has been reported to recognize several amino acid residues in the transmembrane domain of human Tetherin/BST-2 and antagonize the antiviral activity of human Tetherin/BST-2 [29], [30], [31]. The activity of Vpu as a Tetherin/BST-2 antagonist appears to be species specific, since Vpu inhibits the antiviral activity of human Tetherin/BST-2, but not monkey Tetherin/BST-2. In this study, we demonstrated that Vpu inhibited the reduction of RD-114 virus release by human Tetherin/BST-2, but not feline Tetherin/BST-2 (Figure 5). In addition, the cytoplasmic domain of feline Tetherin/BST-2 does not have the STS motif (at position 3-5) required for Vpu/β-TrCP-dependent ubiquitination [32]. Thus, our data support that the activity of Vpu as a Tetherin/BST-2 antagonist is specific for human Tetherin/BST-2.

Similar to human Tetherin/BST-2, the feline homolog is likely to also have antiviral activity against not only RD-114 but also a wide variety of enveloped viruses, although we demonstrated the antiviral activity of feline Tetherin/BST-2 against RD-114. In addition, we showed that the expression of feline Tetherin/BST-2 was induced by type I IFN similar to human Tetherin/BST-2 (Figure 2), suggesting that Tetherin/BST-2 functions as a host innate antiviral system against a wide variety of viruses. Analyses of the expression pattern of feline Tetherin/BST-2 *in vivo* and the mechanism of induction of Tetherin/BST-2 by IFN would be useful for understanding the specificity (tropism) of virus replication in tissues or cells and the development of antiviral strategies against a wide variety of viruses.
